# Continuing the Dialog on Human-Animal Chimerism: Response to Bolo, Wills, and Maschke

**DOI:** 10.1016/j.stemcr.2021.01.003

**Published:** 2021-02-09

**Authors:** Andrew T. Crane, Francis X. Shen, Walter C. Low

**Affiliations:** 1 Department of Neurosurgery, University of Minnesota, Minneapolis, MN, USA; 2 University of Minnesota Law School, Minneapolis, MN, USA; 3 Graduate Program in Neuroscience, University of Minnesota, Minneapolis, MN, USA; 4 Massachusets General Hospital Center for Law, Brain, and Behavior, Boston, MA, USA; 5 Stem Cell Institute, University of Minnesota, Minneapolis, MN, USA

## Main Text

Advances in human-animal chimera embryo (HACE) research have raised a litany of ethical, legal, and social implications (ELSIs) that need to be addressed. Academic research institutions and government organizations, including the National Institutes of Health, have imposed guidelines for how HACE research can be conducted and funded. Organizations such as the International Society for Stem Cell Research published the *Guidelines for Stem Cell Research and Clinical Translation*, which includes recommendations for HACE research with considerations on ethical and animal welfare principles. Similarly, we have previously recommended milestones in HACE research to limit unintended neurological chimerism.

One area that we and our Japanese colleagues believed was overlooked, however, was more diverse stakeholder input beyond simply those who are involved in HACE research. The purpose of our study was to report attitudes of the American public, as a means of better informing policy. A valid concern raised by Bolo, Wills, and Maschke is about the limitation of data collected through the Mechanical Turk platform. In our article, we specifically acknowledge the limitations of the platform and that our survey was “under-powered and not sufficiently diverse to generalize to the American population as a whole.” We also recognized the platform’s limited ability to monitor engagement in real time and sufficiently isolate responses of engaged individuals. While we acknowledge these limitations, we believe that the results of this survey provide a solid foundation upon which discussions involving the ELSIs of HACE research can advance. We encourage other researchers to gather additional data, with larger samples, to further contribute to this discussion.

Bolo, Wills, and Maschke also raise an important point regarding the heterogeneity of HACE research. The methodology of generating an organ composed of human cells in a non-human animal is rather complex and requires base knowledge of stem-cell biology, gene editing, and mammalian development to fully appreciate this complexity and its implications. While we wish we could ask survey participants about their acceptance of HACE research in specific applications, to do so was not feasible given the survey time limitations. We acknowledge that public attitudes will likely shift in individual scenarios particularly when the target organs are the brain and reproductive organs, which can be addressed by additional discussions involving ELSIs of HACE research.

Our survey was not designed to ascertain approval on a particular policy proposal, but rather to gain a general sense of how the public views this research trajectory. The 83% of individuals responding that they can personally accept some form of HACE research is noteworthy in signaling that there is good reason to revisit current policy. At the same time, the concerns of the 17% of individuals who find this research unacceptable and those of the 23% of individuals who did not personally accept the full process of generating an organ for transplantation should be further explored and addressed to ensure that HACE research can continue to provide benefits to society as a whole.

